# Conducting Internet-Based Visits for Onboarding Populations With Limited Digital Literacy to an mHealth Intervention: Development of a Patient-Centered Approach

**DOI:** 10.2196/25299

**Published:** 2021-04-29

**Authors:** Rosa Hernandez-Ramos, Adrian Aguilera, Faviola Garcia, Jose Miramontes-Gomez, Laura Elizabeth Pathak, Caroline Astrid Figueroa, Courtney Rees Lyles

**Affiliations:** 1 School of Social Welfare University of California, Berkeley Berkeley, CA United States; 2 Center for Vulnerable Populations University of California, San Francisco San Francisco, CA United States; 3 Department of Psychiatry, Zuckerberg San Francisco General Hospital University of California, San Francisco San Francisco, CA United States; 4 Division of General Internal Medicine, Zuckerberg San Francisco General Hospital University of California, San Francisco San Francisco, CA United States

**Keywords:** digital literacy, digital divide, underserved, patient-centered, digital health, mhealth, intervention, telehealth, COVID-19

## Abstract

**Background:**

The COVID-19 pandemic has propelled patient-facing research to shift to digital and telehealth strategies. If these strategies are not adapted for minority patients of lower socioeconomic status, health inequality will further increase. Patient-centered models of care can successfully improve access and experience for minority patients.

**Objective:**

This study aims to present the development process and preliminary acceptability of altering in-person onboarding procedures into internet-based, remote procedures for a mobile health (mHealth) intervention in a population with limited digital literacy.

**Methods:**

We actively recruited safety-net patients (English- and Spanish-speaking adults with diabetes and depression who were receiving care at a public health care delivery system in San Francisco, United States) into a randomized controlled trial of text messaging support for physical activity. Because of the COVID-19 pandemic, we modified the in-person recruitment and onboarding procedures to internet-based, remote processes with human support. We conducted a preliminary evaluation of how the composition of the recruited cohort might have changed from the pre–COVID-19 period to the COVID-19 enrollment period. First, we analyzed the digital profiles of patients (n=32) who had participated in previous in-person onboarding sessions prior to the COVID-19 pandemic. Next, we documented all changes made to our onboarding processes to account for remote recruitment, especially those needed to support patients who were not very familiar with downloading apps onto their mobile phones on their own. Finally, we used the new study procedures to recruit patients (n=11) during the COVID-19 social distancing period. These patients were also asked about their experience enrolling into a fully digitized mHealth intervention.

**Results:**

Recruitment across both pre–COVID-19 and COVID-19 periods (N=43) demonstrated relatively high rates of smartphone ownership but lower self-reported digital literacy, with 32.6% (14/43) of all patients reporting they needed help with using their smartphone and installing apps. Significant changes were made to the onboarding procedures, including facilitating app download via Zoom video call and/or a standard phone call and implementing brief, one-on-one staff-patient interactions to provide technical assistance personalized to each patient’s digital literacy skills. Comparing recruitment during pre–COVID-19 and COVID-19 periods, the proportion of patients with digital literacy barriers reduced from 34.4% (11/32) in the pre–COVID-19 cohort to 27.3% (3/11) in the COVID-19 cohort. Differences in digital literacy scores between both cohorts were not significant (*P*=.49).

**Conclusions:**

Patients of lower socioeconomic status have high interest in using digital platforms to manage their health, but they may require additional upfront human support to gain access. One-on-one staff-patient partnerships allowed us to provide unique technical assistance personalized to each patient’s digital literacy skills, with simple strategies to troubleshoot patient barriers upfront. These additional remote onboarding strategies can mitigate but not eliminate digital barriers for patients without extensive technology experience.

**Trial Registration:**

Clinicaltrials.gov NCT0349025, https://clinicaltrials.gov/ct2/show/NCT03490253

## Introduction

### Background

The COVID-19 pandemic has drastically changed how health care is delivered, resulting in an increasing shift to digital and telehealth approaches. The pandemic has also substantially altered patient-facing research, relying on similar digital outreach and implementation strategies [1,2]. Many of these changes to practice and research will likely be sustained into the future even after the pandemic ends. 

Despite the ubiquitous integration of technological innovations into health care, it is important to recognize the inequitable access to digital health tools among marginalized populations. Notably, previous studies show that reduced access to sufficient internet or data plans and low computer use remain barriers to participation in digital strategies among racial or ethnic minorities with low socioeconomic status (SES) [3-5]. Additionally, research has shown that even when access to internet devices and data exists, disparities in usage persists. Specifically, although previous studies have reported high rates of smartphone ownership among minoritized racial or ethnic populations [6], populations of lower SES are more likely to have lower digital literacy [7]. Limited digital literacy has been defined as not having the digital skills necessary to use and navigate internet-driven technology and/or being less comfortable or willing to use digital tools [8-10]. Lower access to digital devices and home internet, combined with limited digital literacy, has alarming implications, given that vulnerable populations are at greater risk for contracting COVID-19 and, therefore, have had to rely on digital and telehealth strategies throughout this pandemic [11].

As patient-facing research transforms into digitized clinical trials, it is important to adopt patient-centered models to ensure recruitment of diverse populations and equitable access to digital health tools for all. Patient-centeredness is defined as “care that is respectful of and responsive to individual patient preferences, needs, and values” and provides patients with the “tools and support they need” [12] to engage in the delivery of their health care [13]. Adopting a patient-centered model of care that focuses on providing patients with the tools necessary to be able to access health care via telehealth strategies would allow research teams, as well as clinicians, to adequately develop generalizable guidelines for conducting internet-based remote visits among vulnerable populations. As health care systems increasingly rely on digital interventions (from telemedicine and beyond), their need to understand the digital profiles of individual patients also increases, especially to improve diverse patient experience.

### Objectives

Through our experience of recruiting English and Spanish speakers of lower SES into clinical research in a public health care delivery system, we developed practical guidelines for internet-based remote recruitment of populations who have access to technology but face digital literacy barriers to self-enroll into mobile health (mHealth) interventions. This paper explains the development process and preliminary acceptability of altering our study procedures from previous in-person sessions to internet-based remote visits for the recruitment of patients with diabetes and depression into an mHealth intervention. We also conducted a preliminary examination of how the composition of recruited patients might have changed from pre–COVID-19 to COVID-19 enrollment periods.

## Methods

### Overview of the mHealth Intervention

The data herein are part of a larger randomized controlled trial (RCT). The Diabetes and Depression Text Messaging Intervention (DIAMANTE) study (NCT03490253) is testing a smartphone-based mHealth intervention (ie, a text messaging system that uses machine learning to personalize content) [14], aiming to increase physical activity among patients with type 2 diabetes and depression and lower SES backgrounds. Patients enrolled in the RCT are sent text messages for a 6-month period. We aim to recruit 276 English- and Spanish-speaking patients aged 18-75 years from a public health care system in San Francisco, USA. This public health care system typically serves patients who are publicly insured and are of low-income status. Interested patients are excluded if they do not own a smartphone or present suicidal ideation and/or active or severe psychosis. Patients are recruited through phone calls following a provider referral and are scheduled for a baseline interview session where they provide consent, answer demographic and relevant questionnaires, and downloaded the DIAMANTE application with support from research assistants. Participants are paid US $40 at baseline and US $70 at the 6-month follow-up. The study was approved by the Institutional Review Board Committee at the University of California, San Francisco (IRB: 17-22608). Prior to the COVID-19 pandemic, we conducted in-person sessions during the usability testing phase and the earlier phase of the RCT (n=32), from January 2019 to March 2020. During this period of in-person interaction with the study participants, our team leveraged existing digital research tools to streamline recruitment and improve retention. We collected measures using Qualtrics surveys [15] rather than paper-based questionnaires; documented patient health information on Research Electronic Data Capture (REDCap)—a secure, web-based software platform [16,17]; and then implemented remote patient monitoring with HealthySMS, a text-messaging platform developed by Dr Adrian Aguilera and approved by the Health Insurance Portability and Accountability Act (HIPAA). HealthySMS has shown high acceptability and engagement among populations of lower SES [18-20]. Each of the above steps were completed in-person during the pre–COVID-19 period, allowing the research assistant to administer face-to-face online Qualtrics assessments or download the intervention app for participants if they were not able to use these platforms on their own.

However, social distancing due to the COVID-19 pandemic necessitated the transition to remote recruitment strategies, without the ability to troubleshoot onboarding issues in-person (eg, assistance with app downloads). To be as efficient as possible in addressing any challenges our target population might encounter in providing consent to participate via DocuSign (rather than via pen-and-paper), completing the online Qualtrics baseline enrollment assessments, and downloading the smartphone application remotely, we significantly altered our study procedures for the screening and recruitment of eligible patients.

### Conceptual Framework

Patient-centeredness has traditionally been used to improve health equity within research and operational programs. In the context of mHealth, patient-centeredness is rooted in active collaboration between patients and research staff [12]. Patients provide insights on unique challenges they face in adopting study procedures, how comfortable they are with trying new modalities, and the level of social support available to them to circumvent challenges. Research staff can leverage their training and expertise to successfully integrate university-mandated measures, processes to increase access to the intervention, and create the infrastructure to offer technical assistance. As such, researchers have the duty to account the demographics of the target population and barriers or facilitators among participants when designing study procedures.

### Approach and Development of Remote Study Procedures

The adaptation of previous in-person study procedures to remote practices took place from March to April 2020. As an initial step, we analyzed the digital profiles—including digital literacy and device or broadband access—of patients who participated in our previous pre–COVID-19 in-person sessions (hereafter referred to as the pre–COVID-19 cohort). Following the analysis, research staff met to discuss personal experiences encountered during previous in-person onboarding sessions. Analysis and feedback allowed us to address the most relevant barriers to and facilitators of an internet-based remote visit. The results led us to alter our study procedures to orient patients to download the digital health application being studied via an internet-based remote visit (ie, by using the Zoom video conferencing software or during a phone call). The project coordinator conducted several educational meetings to introduce and share the rough outlines of the new study procedures with the entire research team. Several amendments were made to these outlines based on onboarding research staff’s feedback and internal piloting efforts.

The final versions were then used to enroll eligible patients (n=11) into a complete, digitized version of the clinical trial during the period of social distancing in the context of the COVID-19 pandemic (hereafter referred to as the COVID-19 cohort), from April 2020 to September 2020. Finally, the enrolled patients who were able to attend a remote onboarding visit were asked about our new remote recruitment processes, and their feedback was both audio-recorded and documented with detailed field notes.

### Measures

Using the online Qualtrics surveys conducted during the usability testing phase and the RCT phase of the DIAMANTE study, we collected several measures to conduct a preliminary examination of how the composition of the recruited sample might have changed from the pre–COVID-19 to the COVID-19 enrollment period.

First, we measured the participants’ SES using the MacArthur Scale. This scale captures the cumulative influences of social hierarchy (including income, education, and occupation) and has been shown to better predict health and wellbeing [21-23]. The description of the MacArthur scale explicitly references objective indicators of SES such as income, education, and occupation. Participants were asked to rate where they perceive their relative position in society on a scale of 1 to 10, wherein 10 signifies the highest SES.

We also measured the patients’ digital literacy. Digital literacy, defined as “the ability to use emerging information and communication technologies to find, access, create, download and communicate information” [24]. Notably, there is no gold-standard measure of digital literacy [25]. Previous studies have measured digital literacy in different ways, including self-reported assessment of perceived skills, with response items ranging from “very poor” to “excellent”; these assessments heavily rely on exploring an individual’s self-efficacy [26-28]. In the context of mHealth interventions, digital literacy involves technical skills (eg, knowing how to use the device) and navigational skills (eg, knowing how to navigate to the App Store) [24]. For our analysis, we considered the conceptual domains of technical and navigational skills and operationalized them. We created our own variable using two multiple choice questions developed by the research team. Participants were asked to rate their answers to the question “How difficult is it to use your smartphone [AND] install mobile applications/apps on your smartphone without someone else helping you?” on a Likert scale of “very difficult,” “somewhat difficult,” or “not difficult.” For our analysis, a patient answering *very* or *somewhat difficult* on both questions was considered to have limited digital literacy.

Finally, semi-structured interviews were conducted to explore the acceptability of remote onboarding procedures. Questions were based around downloading the app and the experience during and after completion of an internet-based remote session for clinical research.

### Data Analysis

Given the small sample size, nonparametric, paired-sample, one-sided Fisher exact test was performed using SPSS (version 27.0; IBM Corp) software to capture differences in digital literacy scores between the pre–COVID-19 and COVID-19 cohorts. We considered a difference to be statistically significant at *P*<.05.

To explore acceptability of the new onboarding procedures, we categorized enrolled participant feedback about the remote onboarding procedures from our field notes and audio-recorded interviews. RH read the interviews in their entirety, using the semistructured format of the interview guides to code for the categories represented in the interview guide as follows: (1) overall experience with an internet-based remote session and (2) difference and benefits or drawbacks of an internet-based remote visit in comparison to a patient-facing visit.

## Results

### Study Characteristics

Table 1 summarizes overall characteristics of the study participants. Their mean age was 52.8 (SD 10.5) years. Of the 43 participants, 24 (55.8%) were female; 38 (88.4%) were non-White participants; 20 (46.5%) were Spanish-speaking participants; 30 (69.7%) had a high-school education or lower; and 29 (67.4%) were unemployed, retired, or on disability.

**Table 1:** Baseline demographic characteristics.

Self-reported characteristic			Overall (N=43)		Pre–COVID-19 cohort (n=32)		COVID-19 cohort (n=11)
Age (years), mean (SD)			52.8 (10.5)		53.6 (9.45)		50.5 (0.6)
Socioeconomic status (score), mean (SD)			4.66 (2.30)		4.79 (2.11)^a^		4.36 (2.77)
**Interview language, n (%)**							
	Spanish	20 (46.5)		18 (56.3)		5 (45.5)	
	English	23 (53.5)		14 (43.8)		6 (54.5)	
**Sex, n (%)**							
	Female	24 (55.8)		20 (62.5)		4 (36.4)	
	Male	19 (44.2)		12 (37.5)		7 (63.6)	
**Ethnicity, n (%)**							
	White	4 (9.3)		3 (9.4)		1 (9.1)	
	Black	4 (9.3)		3 (9.4)		1 (9.1)	
	Hispanic or Latino(a)	26 (60.5)		20 (62.5)		6 (54.5)	
	Asian or Pacific Islander, or other	9 (20.9)		6 (18.8)		3 (27.3)	
**Marital status, n (%)**							
	Single	18 (41.9)		12 (37.5)		6 (54.5)	
	Married or partnered	9 (20.9)		6 (18.8)		3 (27.3)	
	Divorced or legally separated	13 (30.2)		12 (37.5)		1 (9.1)	
	Widow	3 (7.0)		2 (6.3)		1 (9.1)	
**Education, n (%)**							
	High school or lower	30 (69.7)		23 (71.9)		7 (63.6)	
	More than high school	13 (30.2)		9 (28.1)		4 (36.4)	
**Employment status, n (%)**							
	Disabled or on disability	17 (39.5)		14 (43.8)		3 (27.3)	
	Part-time or more	14 (32.6)		10 (31.3)		4 (36.4)	
	Unemployed	10 (23.3)		8 (25)		2 (18.2)	
	Retired	2 (4.7)		0 (0)		2 (18.2)	

^a^Results for this question are missing data from 8 patients who participated in the usability testing phase of the project and were not provided with this measure.

### Socioeconomic Status

In all, 35 of the 43 (81.4%) participants filled out the MacArthur Scale, including all RCT participants. Overall, our sample self-identified as having low SES (mean 4.66, SD 2.30). Specifically, the self-reported mean score for patients in the pre–COVID-19 cohort was 4.79 (SD 2.11), whereas the mean score for those in the COVID-19 cohort was 4.36 (SD 2.77).

### Changes to Study Procedures

Multimedia Appendix 1 details the pre–COVID-19 (in-person) versus the current COVID-19 (remote) recruitment and onboarding procedures. Broadly, these steps outline our implementation of brief, one-on-one staff-patient interactions to provide technical assistance personalized to each patient’s digital literacy. More specifically, we rolled out multiple new procedures. First, we prepared for the remote onboarding visits: our team developed educational materials, such as YouTube videos on how to download the Zoom app onto a smartphone device, written brochures on how to access an email or sign with DocuSign using a smartphone device, and a manual on how to download the DIAMANTE mobile app. Then, during the recruitment visits, we sent real-time SMS links for app downloads and used a video conferencing software (eg, Zoom) for initiating communication on the onboarding session. If we were successful with the Zoom call for the onboarding session, we were then better able to establish a rapport with the participant despite the remote environment. This allowed us to be able to see the participant’s digital devices better, which facilitated troubleshooting of any technical issues. In the event that a video call via Zoom was not possible, the staff learned more details about Android and iOS operating systems to assist with common technical problems and be able to verbally explain the various steps involved (in both English and Spanish versions of the operating systems). Finally, patients who seemed hesitant (either in their ability to set-up for a Zoom session and/or in downloading the smartphone app based on remote instructions) requested that their tech-savvy loved ones be present during the onboarding session. Staff then worked with these patients and their loved ones and ensured that both of them understood every step of the process.

### Digital Profiles

After implementing these procedures, we then compared the digital access and literacy among participants in the pre–COVID-19 cohort with those in the COVID-19 cohort (Table 2). Although we enrolled participants who were racially or ethnically diverse and had lower SES in both time periods, we had a higher recruitment rate of individuals with access to Wi-Fi within the COVID-19 cohort. A total of 18 of the 32 (56.3%) participants in the pre–COVID-19 cohort had access to Wi-Fi at their home, compared to all 11 (100%) participants in the COVID-19 cohort. Primary outcome results indicated a nonsignificant reduction in the self-reported digital literacy between the cohorts, with 34.4% (11/32) of the participants in the pre–COVID-19 cohort reporting limited digital literacy as compared to 27.3% (3/11) of the participants in the COVID-19 cohort (*P*=.49).

**Table 2:** Patients’ digital profiles.

Self-reported characteristic			Overall (N=43)		Pre–COVID-19 cohort (n=32)		COVID-19 cohort (n=11)	
**Digital access, n (%)**								
	Wi-Fi at home	29 (67.4)		18 (56.3)		11 (100)		
**Smartphone type, n (%)**								
	Android	27 (62.8)		18 (56.3)		9 (81.8)		
	iPhone	16 (37.2)		14 (43.8)		2 (18.2)		
**Digital literacy, n (%)**								
	Difficulty using smartphone	18 (41.8)		14 (43.8)		4 (36.3)		
	Difficulty installing apps	22 (51.2)		18 (56.3)		4 (36.3)		
Interviewer downloaded the app for the participant			32 (74.4)		32 (100)		0 (0)	

### Patient Experience

Finally, we summarized patient experiences with the new remote onboarding procedures. In all, 8 of 11 (73%) patients in the COVID-19 cohort had never participated in a remote visit for research in the past, but all 11 (100%) patients stated that they enjoyed participating in remote visits for research. One patient, for example, expressed that staff taking the time to offer technological assistance allowed them “*to see the hospital's commitment to the community.*” In addition, 7 of 11 (64%) patients classified time efficiency and the convenience of not having to travel outside their home as a major benefit to participating in research via remote visits.

When discussing how this research study fit into their overall needs for digital health care at our safety-net health care setting, the patients remained supportive of these remote approaches during the COVID-19 pandemic and beyond:

It's still important to have someone guide patients for things that are confusing in this remote session. I felt safe doing this.

The technology for video conferencing is overdue, and we should have been doing it for a longer time, but now we have been forced to adapt…[it] feels wonderful to be included in all… Video conferencing can be used to reach out to populations or cultures that have a harder time going to their appointments.

Despite these positive comments, 3 of 11 (27%) patients stated that they would still prefer an in-person patient-facing session in the future, given the simplicity of steps involved.

## Discussion

### Principal Findings

With the speedy uptake of digital and telehealth strategies propelled by the COVID-19 pandemic, it is a high priority to proactively reform current practices to ensure equitable access among vulnerable populations, including those who might have access to technology and broadband but face additional digital literacy barriers. Prior to the COVID-19 pandemic, the majority of health care systems did not provide training for populations with limited digital literacy to ensure adoption of the available digital health tools, including mHealth interventions and telehealth or telemedicine [29-31]. To address this challenge, our research team altered the screening procedures and developed a new onboarding protocol that centrally considered a patient’s digital literacy skills. This resulted in the development of one-on-one staff-patient partnerships and a multitude of resources for users and staff to circumvent challenges in accessing a new modality of engagement instead of the previous face-to-face research participation approach. In developing and piloting new onboarding procedures, we leveraged several implementations strategies, including conducting educational meetings with onboarding research assistants, development of educational materials for users and staff, identification of early adopters, and building expertise with participant facilitation via phone and Zoom calls. A patient-centered approach allowed us to anticipate possible challenges our target population might face and proactively prepare strategies or materials to circumvent such challenges. For example, early on, we knew that there was a need to develop steps that accounted for smartphone software given the many technical differences that exist between Android and iOS interfaces. In addition, we also personalized the level of human support provided in our study by using our digital access and literacy questions to offer the right type of assistance.

Furthermore, we started data collection to evaluate whether patients of lower SES and limited digital literacy were still able to participate in mHealth research in the midst of direct economic and health consequences of the COVID-19 pandemic. Our findings indicate that one-on-one staff-patient partnerships can be effective in providing technical assistance to populations who have limited digital literacy skills and are interested in gaining access to digital health tools. We were able to enroll a sample similar to our pre–COVID-19 cohort during the COVID-19 social distancing period by using a new onboarding procedure. Specifically, although, there was a nonsignificant reduction in patients with limited digital literacy enrolling into our study during the COVID-19 pandemic, both cohorts had challenges with digital literacy. The COVID-19 cohort had unique challenges in trying to use both our DIAMANTE app as well as Zoom (many participants were using these apps for the first time) without the assistance of in-person support (eg, research assistants assisting participants to download the app if they were not able to use these platforms on their own). Our findings demonstrate that with adequate attention and support, it is possible to reach patients with limited digital literacy by using remote strategies. This finding is consistent with the previous literature that shows that patients of lower SES have high interest in using digital platforms to manage their health, but they may require additional upfront human support to gain access and have an overall better user-experience [32,33]. At the same time, it is important to recognize that this is not a one-size-fits-all approach. Importantly, 3 of 11 (27%) patients that enrolled in our study using the new screening and onboarding procedures indicated they preferred in-person sessions as opposed to an internet-based remote session for research enrollment.

Although our rate of recruitment has slowed down overall given the pandemic, we know that patients are able to enroll into the study and successfully download a smartphone app through visual and verbal coaching from research assistants. Patients showed willingness to participate in the development of procedures, provided feedback on how to improve user-experience, and ultimately, showed acceptability of internet-based remote sessions for research.

### Limitations and Comparison With Prior Work

Previous research reveals gaps in accessibility to telehealth, telemedicine, and remote strategies within vulnerable populations, particularly those with lower technical abilities, older age, and limited English proficiency—these issues are particularly acute in the context of the COVID-19 pandemic [34,35]. Other work has specifically called for researchers working with vulnerable populations to consider different participant profiles and target individuals’ needs [36,37]. As this preliminary study was conducted with a small sample size and only in San Francisco, our results might not be generalizable to other settings. We did not perform statistical comparisons of our remote onboarding approaches, but we expect to gain and report more data as the DIAMANTE RCT continues. Moreover, we believe the enumeration of protocol adaptations made in this study increases the concrete tactics for others working with individuals with limited digital literacy on telehealth and digital health interventions in general.

### Conclusions

The COVID-19 pandemic has emphasized that we must identify clear strategies for engaging patients with limited digital literacy to extend health care services (and access to research studies) outside of in-person care. Engagement strategies in clinical settings are necessary in order to prevent further growth of health disparities. In addition, ensuring engagement in digital and remote research will ensure an inclusive knowledge base.

Responses to expanding the use of technology in care have included policy changes to expand reimbursement for telemedicine, and efforts to expand access to the internet. In addition to these priorities, our findings indicate the need for additional research and practice to address digital literacy barriers and ensure equitable access to digital health care interventions in the near and long term. Researchers who focus on digital health interventions should prioritize better reporting of digital literacy capabilities of their enrolled participants, incorporate service design frameworks within their trial protocols, and thoroughly explain the steps needed to enroll in digital health interventions. Implementation techniques across digital health trials should be leveraged to facilitate the translation of research findings into clinical practice. For example, health care systems could implement brief screenings prior to remote visits to know whether a patient is ready to try a video visit or needs additional support or training. The guidelines we have developed can benefit clinicians and clinical researchers working with vulnerable patients with limited digital literacy to ensure that digital advancements in health care do not lead to increased health disparities. Moving forward, we must dedicate attention and practical resources to those with lower levels of digital literacy as we continue to rapidly expand access to digital health tools; otherwise, we risk increasing existing health disparities for both telemedicine uptake as well as broader health care and research interventions. 

## Appendix

Multimedia Appendix 1 Comparison of recruiting and onboarding practices for enrolling patients with limited digital literacy onto a digital health intervention during the pre–COVID-19 and COVID-19 periods.

Multimedia Appendix 2 CONSORT-eHEALTH checklist (V 1.6.1).

## Abbreviations

DIAMANTE: Diabetes and Depression Text Messaging Intervention
HIPAA: Health Insurance Portability and Accountability Act
mHealth: mobile health
RCT: randomized controlled trial
REDCap: Research Electronic Data Capture
SES: socioeconomic status
