# Supplementary material for: Conducting Internet-Based Visits for Onboarding Populations With Limited Digital Literacy to an mHealth Intervention: Development of a Patient-Centered Approach
Source: JMIR Form Res. 2021 Apr 29;5(4):e25299. doi: 10.2196/25299 (PMC8086779; doi:10.2196/25299)
Supplement: Multimedia Appendix 1 [file formative_v5i4e25299_app1.docx]

**Multimedia Appendix 1.** Remote practices for enrolling patients onto a digital intervention

| **Digital Assessment** | **Pre-COVID** | **COVID** |
| --- | --- | --- |
| **Recruitment Screening** | *One telephone call:*   - Research assistant determines eligibility criteria (e.g. patients with depression and diabetes who do not have serious physical limitations and who own a smartphone) - If eligible, research assistant determines: - Patient’s smartphone software (iOS/ Android) | ****Can take multiple telephone calls:*   - Research assistant determines eligibility criteria (e.g. patients with depression and diabetes who do not have serious physical limitations and who own a smartphone) - If eligible, research assistant determines: - Patient’s accessibility to at-home Wi-Fi - Patient’s smartphone software (iOS/ Android) - Patient ability log-in to their email and whether they know their Apple ID/Google Play store and password - Patient’s accessibility to multiple smart devices (e.g. other smartphone, computer or tablet) - Patient’s accessibility to outside technical help from a loved one (for troubleshooting) - Based on answers, research assistant schedules a remote visit via a phone call or video call via Zoom |
| **Baseline Visit** | *In-person visit*   - Paper-based consent forms signed at baseline visit with research assistant present - Baseline survey completed via Qualtrics through iPads provided to patients during scheduled visits - Smartphone application for mHealth intervention downloaded by staff or with staff assistance | *Telephone or Zoom visits*   - Email consent forms via DocuSign prior to baseline appointment - Send Zoom meeting ID and password via text, email or through verbal diction (phone call) - Research assistant is available with assistance to facilitate Zoom download/log-in via a phone call - Research assistant sends Qualtrics survey link via text or email to participant - Survey completed by participant or with research assistant’s assistance via a Zoom shared screen or over the phone - Research assistant sends direct download link of the smartphone application via text while providing technical verbal/visual instructions to complete app enrollment |
